# Effects of Gut Microbiota on Host Adaptive Immunity Under Immune Homeostasis and Tumor Pathology State

**DOI:** 10.3389/fimmu.2022.844335

**Published:** 2022-03-10

**Authors:** Yanan Li, Zixuan Ye, Jianguo Zhu, Shuguang Fang, Lijuan Meng, Chen Zhou

**Affiliations:** ^1^ School of Food Science and Pharmaceutical Engineering, Nanjing Normal University, Nanjing, China; ^2^ Research and Development Department，Wecare-bio Probiotics Co., Ltd., Suzhou, China; ^3^ Department of Oncology, First Affiliated Hospital of Nanjing Medical University, Nanjing, China; ^4^ Phase I Clinical Trial Unit, The First Affiliated Hospital of Nanjing Medical University, Nanjing, China

**Keywords:** microbiota, adaptive immunity, homeostasis, tumor, immunotherapy

## Abstract

Gut microbiota stimulate and shape the body’s adaptive immune response through bacterial components and its active metabolites, which orchestrates the formation and maintenance of the body’s immune homeostasis. In addition, the imbalances in microbiota-adaptive immunity contribute to the development of tumor and the antitumor efficiency of a series of antitumor therapies at the preclinical and clinical levels. Regardless of significant results, the regulation of gut microbiota on adaptive immunity in immune homeostasis and tumors needs a more thorough understanding. Herein, we highlighted the comprehensive knowledge, status, and limitations in the mechanism of microbiome interaction with adaptive immunity and put forward the prospect of how to translate these insights in inhibiting tumor progression and enhancing the efficacy of antitumor interventions.

## 1 Introduction

The human gastrointestinal tract is a highly evolved human–microbe interface, which was colonized by a tremendous number of microorganisms, especially 10^11^−10^12^ microorganisms per cm^3^ in the colon (1). In health, gut microbiota and the host have formed a carefully negotiated, mutually beneficial, relatively stable, and delicately balanced symbiont (2–6). Indeed, the diverse and abundant gut microbiota is vital to promoting the evolution and maturity of innate immunity and adaptive immunity through its continuous stimulation and education in homeostasis(7–10). In addition, the mucosa has become highly adapted to the presence of commensal bacteria, although the complete mechanism is still mostly undiscovered (3, 11). Interestingly, the perturbation of the gut microbiome by gene drivers or environmental factors often results in the aberrant immune responses, which is implicated in the development of malignant tumors. With the increasing incidence of malignant tumors, studies have confirmed that intestinal bacteria also profoundly shape the host’s antitumor immunity (12). Among the immune system, the ancient innate immune system is essential to obliterating many pathogens and transformed cells *via* identifying pathogen-associated molecular pattern (PAMP) and damage-associated molecular pattern (DAMP). However, the range of PAMP and DAMP is limited that pathogens and transformed cells have the ability to change their molecular models to evade innate immune mechanisms as well. Therefore, the innate immune system needs to activate and cooperate with adaptive immunity to enhance complete immune responses (13, 14). Moreover, the overwhelming variability of bacterial antigens has driven the evolution and specific memory of the adaptive immune system which enables the rapid response to previously encountered pathogens(13, 15). Other reviews have reviewed the aspects of the interaction between intestinal microbiome and innate immunity (8, 16). Based on the perspective that commensal bacteria themselves are also immunogenic, our review focused on the education and regulation of gut microbiota on adaptive immunity. Here, we review the key concepts and insights linking the microbiota to the activation and function of adaptive immunity under immune homeostasis and tumor pathology state. Based on the microbiota-adaptive immunity interaction mechanism, we further highlighted the potential of microbiota in the intervention strategy of antitumor fields.

## 2 Gut Microbiota Regulates Host Adaptive Immunity Under Homeostasis

The main members of the adaptive immune system include humoral immunity mediated by B cells (the source of antibodies) and cellular immunity mediated by T cells (17). The specificity and diversity of T cell receptor (TCR) and B cell receptor (BCR) repertoires are the core characteristics of adaptive immunity (18). Due to the wide variety of antigens carried by the intestinal flora, gut microbiota play a key role in promoting the evolution of TCR and BCR (and its antibody) repertoires (19). After antigens from commensal bacteria stimulate adaptive immune cells, the genes encoding TCR and BCR are rearranged and assembled to produce extremely diverse receptors (15, 20). Theoretically, the adaptive immune system with high specificity and flexibility has the potential of recognizing all potential pathogens or transformed cell antigens which further initiate effective adaptive immune response (17). Mutual promotion and restriction between gut microbiota and the adaptive immune are the basis for maintaining immune homeostasis.

### 2.1 Role of Gut Microbiota in Host Immune System Under Homeostasis

The mucosal barrier is composed of tightly connected intestinal epithelial cells (EC) which is covered with a mucin-hydrated gel layer including a variety of antibacterial substances such as secretory IgA (sIgA) and antibacterial peptides (21). The intestinal mucosal barrier effectively restricts the penetration of most intestinal microorganisms into the mucus layer (22). As we know, segmented filamentous bacteria and some Bacteroides species (*Bacteroides fragilis* and *Bacteroides thetaiotaomicron*) could go through the mucus layer and localize on the epithelium cells in the small bowels (23) and colonic crypts (24), respectively. *Mucispirillum schaedleri*, as the only genus of *Deferribacteraceae* in vertebrate gut, is the dominant member of cecal crypt microbiome (25). The microorganisms that penetrate into the mucus layer can be presented to immune cells by dendritic cells (DC) or by the endocytosis of microfold cells (26, 27). DCs can carry a small amount of commensal bacteria to mesenteric lymph node (mLN) and stay there for several days (28–30). DC migration from the intestine to the mLN is confined to lymphatic vessels, and usually DCs cannot cross the first draining lymph node they enter (29, 31). Thus, the commensal bacteria remain in the intestinal immune compartment from entering the systemic immune circulation, which effectively guarantees that the commensal bacteria can produce effective mucosal immune response without causing systemic side effects under immune homeostasis (28–30). Besides, the infected EC cells can also be phagocytosed by macrophages to deliver the antigen to activate adaptive immunity. In addition to interacting with the immune system through bacteria itself, the gut bacteria can also influence the immune system through producing active metabolites such as short-chain fatty acids (SCFAs) (32–41), secondary bile acids (BAs) (42–44), and inosine.

### 2.2 Gut Microbiota and Adaptive Humoral Immunity

The crucial mediators of adaptive humoral immunity, such as B cells, could maintain gut homeostasis by producing a large number of sIgA antibodies responsive to commensal bacteria (45). Firstly, microbes are standard immunogens that induce the increase of antigen-specific sIgA. In the presence of commensal bacteria, more than 80% of human plasma cells reside in the intestinal lamina propria and secrete IgAs (46), which accounts for more than 70% of the systemic immunoglobulin production(30). Germ-free (GF) mice harbor a multitude of defects in their immune system (47). When Dennis L. Kasper et al. monocolonized GF mice with each of 53 microbial strains derived from the human gut, they found that the sIgA levels in all stools were between GF mice and specific pathogen-free mice (SPF). There was a significant correlation between total sIgAs and organism-specific sIgAs, suggesting that microbes boost sIgA production as genuine antigens (48). These sIgAs coat commensal bacteria through the affinity between them and then restrict their growth to limit their ability to penetrate the mucosal barrier (49), which further assist host to defense against pathogens and contribute to the immune balance between commensal bacteria and host. Intestinal plasma cells can produce mucosal IgAs through T cell-dependent (TD) and T cell-independent (TI) mechanisms, among which most commensals elicited strong TI responses. A minor series of atypical commensal bacteria, including segmented filamentous bacteria (SFB) and *Mucispirillum*, escape the TI response due to lack of sites for TI antigen-induced sIgAs. After they penetrate the mucus layer, they mainly interact with antigen-presenting cells and trigger adaptive B cell and T cell responses to produce antigen-specific sIgAs (46). Besides, commensal bacteria with flagella could also promote sIgA production through stimulating Toll-like receptor (TLR) 5 on DCs by flagellin in the lamina propria (LP) which further activated naïve B cells to differentiate into IgA-producing plasma cells (50). Humans with defective IgA secretion have increased susceptibility to inflammatory bowel disease, celiac disease, and allergies (51, 52).

It is worth noting that sIgA coating of commensal bacteria mainly targets the small intestinal flora instead of the colonic bacteria due to their different anatomical structures (46). Among that, Peyer’s patches (PPs) and isolated lymphoid follicles are mainly related to the small intestine, and the small intestines include special epithelial cells that can present luminal antigens. Although some sIgA-coated commensal bacteria in the colon are also detected, it may be the interference by the upstream small intestine flora (46). Until now, the significance of the different sIgA coating of colon and small intestine commensal bacteria is currently unclear in homeostasis.

In addition to intestinal sIgAs, IgM and some IgG subclasses are also associated with the intestinal microbiota in the intestinal homeostasis state. There also existed IgM-secreting cells on the gut mucosa that is clonally related to IgM-memory B cells in human (53), which release sIgM into the gut lumen through the polymeric Ig receptor (54). When the immune exclusion is induced by sIgAs, studies have evidenced that sIgMs could bind certain pathogens, e.g., *Salmonella enterica Typhimurium*, and then limit their entry into Peyer’s patches (55). In addition, sIgMs are also found to modulate the expression of bacterial virulence factors to preserve epithelial surface homeostasis (56). Besides, Magri et al. demonstrated that sIgMs optimize microbiota diversity in ileum and colon *via* coating commensals, which may maximize mucus retention of specific bacterial communities with benefits (57, 58). Recently, a robust level of serum IgGs that could bind commensal bacteria is also detective in healthy humans (59–62). Although the intestinal B cells are dominated by IgA-producing cells, 3%–4% of intestinal antibody-secreting cells express IgGs at steady state (63, 64), whose mechanism needs to be further investigated. In summary, the humoral regulation by commensal bacteria may represent a dominant force that maintains the evolution of the peripheral B cell lineage to promote its diversification.

### 2.3 Gut Microbiota and Adaptive Cellular Immunity

**Figure 1:**
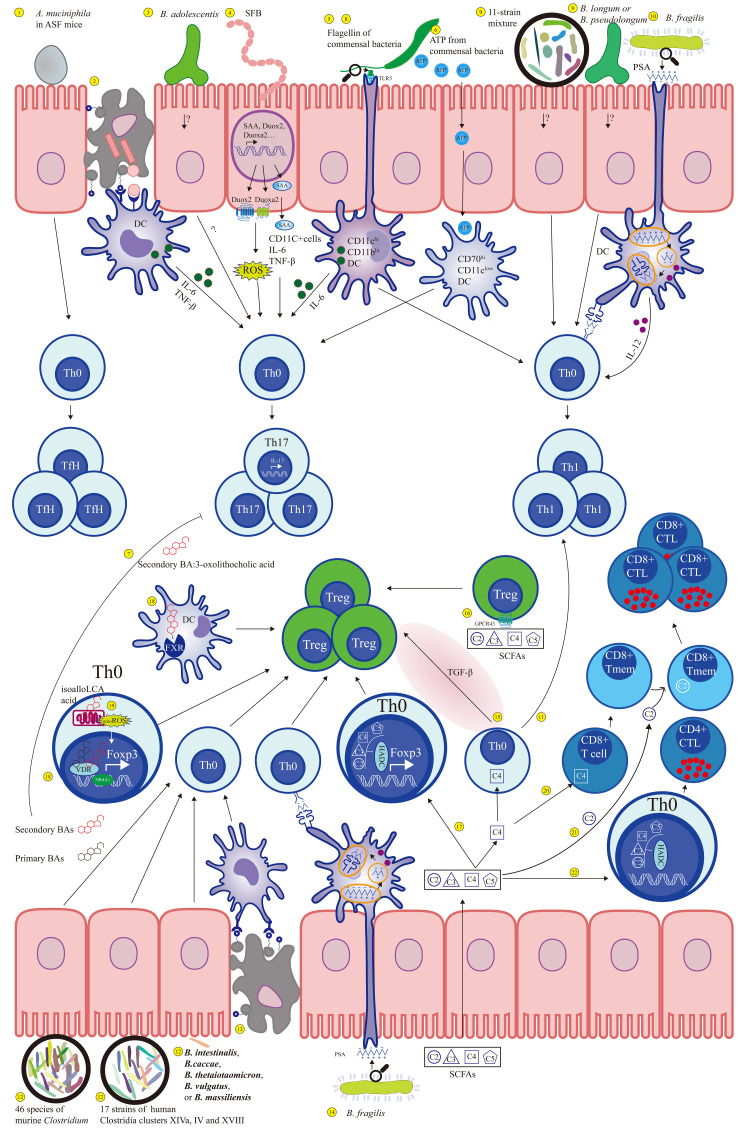
Gut microbiota affects adaptive immunity under physiological conditions through a series of immune signal. 1. *Muciniphila* can stimulate TH0 cells to differentiate into TfH cells. 2–6. Symbiotic bacteria promote Th17 differentiation through a series of immune signal mediations. 7. The metabolites of bacteria, 3-oxolithocholic acid (secondary BA) inhibit Th17 cell differentiation. 8–11. Flagellin of symbiotic bacteria, symbiotic bacteria, and their metabolites can promote Th1 differentiation. 12–19. The differentiation of Treg can be induced by symbiotic bacteria, their mixture and metabolites. 20. Butyrate can enhance the memory effect of CD8+ T cells. 21. Increased acetic acid concentration is integrated by CD8+ T cell function, promoting rapid memory CD8+ T cell response. 22. SCFA treated with CD4+ T cells as an HDAC inhibitor can upregulate expression of CTL gene and increase CD4+ T cells.

Studies during the past decade have provided a more complex picture of the cross talk between the gut microbiome and cellular immunity (65–67). In cellular immunity, adaptive T cells are the main members that defend the homeostasis of the host from suffering from immune-mediated inflammatory diseases (**Figure 1**). The intestinal microbiome could promote the differentiation of T cells to quickly respond to the signal from the intestinal lumen environment and initiate adaptive immune responses (68).

#### 2.3.1 Regulation of Gut Microbiota on CD8+ T Cells

CD8+ T cells, distinguishing infected or diseased cells from healthy cells, are pivotal in antiviral and antitumor immunity (68). Studies have evidenced that most human-derived monoclonal intestinal bacteria find it difficult to disturb the level of CD8+T cells after colonizing GF mice (48). However, a specific strain, *Fusobacterium varium*, significantly disturbs the intestinal adaptive immune phenotype after monoclonal colonization. *F. varium* significantly reduced the populations of CD4+ T cells and CD8+ T cells and caused a higher frequency of colonic double-negative cells (CD4−CD8−TCRβ+) than other microbes. Besides, *F. varium* strongly suppressed a large group of genes related to bile acid metabolism, which has been shown to be closely related to immune function (48). Another study showed that the 11-strain mixture acted as a consortium to stimulate the immune system in which the 4 non-Bacteroidales species act as effector elements and other 7 Bacteroidales as supporting elements (69). These strains were identified by CD103+ dendritic cells in an MHC-Ia-dependent manner, leading to accumulation of IFNγ+ CD8 T cells in the intestinal LP, which significantly disturbed the cellular immune function. Most of the colonic IFNγ+ CD8+ T cells are differentiated from conventional CD8+ T cells, such as activated T cells, memory T cells, and cytotoxic T cell subsets, which display relative enrichment of TCR Vβ6+ and Vβ8+ subsets to specifically recognize bacterial antigens. Besides, no colonic inflammation was observed in mice at the histological level, indicating that the 11-strain mixture positively modulates CD8 + T cell activity in a relatively specific, safe manner under the immune homeostasis state (69).

#### 2.3.2 Regulation of Gut Microbiota on CD4+ T Cells

Adaptive immune responses highly rely on differentiation of CD4+ T helper cells into subsets with diverse effector functions best suited for host defense against invading pathogens such as Th1s, Th2s, Th17s, Tfhs, iTregs, and other subtypes (70, 71). The balance between these subtypes is an important factor in maintaining immune homeostasis.

##### 2.3.2.1 Regulation of Gut Microbiota on Th17s

Th17s are highly proinflammatory T cells that are involved in immune clearance against extracellular bacteria and fungi (72, 73), playing a critical role in the intestinal mucosal immune defense system. Th17s also trigger B-cell proliferation and promote antibody production through class switch recombination (74), which is vital in the formation of ectopic lymphoid follicles and tertiary lymphoid follicle-like structures in target organs (75–77). Generally, the synergy between transforming growth factor-β (TGF-β) and IL-6 induces Th17 differentiation, and IL-23 supports the expansion and maintenance of Th17 cells (78).

Gut microbiota contributes to the constitutive development of Th17s cells in the LP through a variety of mechanisms (79, 80). Firstly, the recognition and phagocytosis of infected apoptotic cells by DCs uniquely trigger the combination of IL-6 and TGF-β to promote Th17 differentiation (81). The adhesion of commensal bacteria to EC is also a key clue for Th17 induction. Among the symbionts, SFB is one of the most effective Th17 inducers (82, 83). SFB usually penetrates into the mucus layer and adhere to EC, leading to the highly upregulated expression of some genes in ECs, such as serum amyloid A (SAA), the reactive oxygen species (ROS)-generating enzyme dual oxidase 2 (Duox2), and its maturation factor Duoxa2. Under the mediation of CD11c+ cells, IL-6 and TGF-β, SAA significantly enhances the differentiation of naïve CD4+ T cells into Th17s (84). SAA can also directly act on RORγt+ T cells to induce IL-17 expression (85). Th17 differentiation is significantly affected after ROS clearance with a ROS scavenger, N-acetyl-L-cysteine (NAC) (84). Most of Th17s induced by SFB have TCRs that specifically recognize SFB antigens. *Bifidobacterium adolescentis*, common commensal bacteria in the human intestine, is also closely related to producing homologous Th17s, which may work similarly to SFB in humans (86).

In addition, the flagella of commensal bacteria as strong immunogens could stimulate TLR5 expressed on CD11c^hi^CD11b^hi^ LP DCs to induce IL-6 secretion, thereby promoting Th17 differentiation (50). Furthermore, adenosine 5′-triphosphate (ATP) derived from commensal bacteria also leads to Th17 differentiation by activating a subset of CD70^hi^CD11C^low^ DCs in LP through MyD88 and TRIF-independent mechanisms (79). Some human-derived microorganisms such as *Clostridium histolyticum*, *Clostridium sordellii*, and *Acinetobacter baumannii* induce Th17s to levels similar to those driven by SFB (48). As *C. histolyticum*, *C. sordellii*, and *A. baumannii* can cause human infections, the upregulation of Th17s is vital in preventing infection and maintaining homeostasis. Besides, as a double-edged sword, Th17s are also responsible for intestinal inflammation and are associated with autoimmune diseases (87). Other commensal bacteria simultaneously present in the intestine to stimulate Treg differentiation, which are in charge of exerting immune tolerance to avoid excessive inflammation. The complex commensal microbiome dynamically maintains immune homeostasis by balancing the pro-inflammatory Th17s and the anti-inflammatory Tregs (87).

##### 2.3.2.2 Gut Microbiota Boost Th1s Differentiation and Correct Th1/Th2 Skewness Under Homeostasis

Th1s and Th2s mediate distinguished immune response on the basis of their unique expression pattern of cytokines and transcription factors (88). Th1s mainly evoke cell-mediated immunity and phagocyte-dependent inflammation, while Th2s elicit eosinophil accumulation and antibody-induced responses and inhibit several functions of phagocytic cells (89). Viewed from the traditional perspective, the healthiest immune state could be one poised equally between “cellular immunity” (approximating Th1s) and “humoral immunity” (Th2s). In GF mice, the total number of lymphocytes was downregulated, leading to reduced secretion of cytokines. Thus, the ratio of Th1s/Th2s was unbalanced and tilted toward Th2s. Colonization of normal flora or specific commensal strains can correct these immune abnormalities. The polysaccharide A (PSA) of *Bacteroides fragilis* is processed into low-molecular-weight carbohydrates through a nitric oxide-mediated mechanism and presented to T cells through MHC-II by DCs, and then activates Th1 responses (90). *Bifidobacterium longum*, *Bifidobacterium pseudolongum*, and a specific strain mixture can also induce a significant increase of Th1s in small intestinal or colonic LP (48, 69, 91). In addition, commensal bacteria can also stimulate DCs in LPs through flagellin, which further promote Th1 differentiation (50). However, the role of the microbiome in Th1/Th2 balance still requires further research and discovery.

##### 2.3.2.3 Regulation of Gut Microbiota on Tfhs

In addition to effector T-cell subsets, a specialized T helper cell subset, called follicular B helper T cells (Tfh), is essential for B cell activation, isotype class switching, and germinal center formation (92). Most of the intestinal immunoglobulins are produced by B cells in Peyer’s germinal center with the help of Tfhs [81, 82]. To reveal specific immune response, altered Schaedler flora mouse is an important tool for studying microbiome–host interactions. As recorded, the altered Schaedler flora (ASF) is a model community of eight microorganisms that was developed by RP Orcutt and has been in use since the late 1970s (93). Moreover, the ASF+ *Akkermansia muciniphila* (*A. muciniphila*) system produced a commensal specific T cell response mainly limited to Tfhs in the mLN or PPs, with very few Tregs, Th1s, Th2s, or Th17s, indicating that Tfhs can be differentiated from naïve commensal-specific T cells (94). Certainly, the relationship between and Tfhs and microbiota is reciprocal, because Tfh cells are also implicated in the maintenance of microbiota homeostasis as recorded by studies which showed that the impairment of Tfh cells can further alter the gut microbiota composition (95, 96).

##### 2.3.2.4 Regulation of Gut Microbiota on Tregs

Regulatory T cells (Tregs) that expressed the transcription factor (TF) FoxP3 are an important component in restraining the excessive immunoreaction and maintaining immunological homeostasis in the intestines. Tregs target most immune cells to trigger immune tolerance in an antigen-specific or non-specific manner, through contact-dependent mechanisms, immunomodulatory cytokines (e.g., IL-10, TGF-β, and IL-35), or the metabolic disturbance of target cells (97, 98). In the host, Tregs are mainly divided into two different groups. One group includes CD4+CD25+Foxp3+ natural regulatory T (nTregs) cells that differentiate from immature precursor cells in the thymus, and the other one includes induced regulatory T (iTregs) cells that are *de novo* produced by naïve conventional T cells chiefly in the intestinal immune niche (98, 99). Owing to the independent developmental environment, the TCR repertoires of nTregs and iTregs do not overlap in the main body (9, 100–102). Intestinal Tregs are mainly iTregs which were divided into two subsets according to the expression of additional TFs. The first subset was RORγ+ Treg cells which predominate in the colon, with the expression of the nuclear hormone receptor RORγ and TF c-Maf, and are also key regulators for group 3 innate lymphoid cells and Th17 cells (103, 104). The second subset that expresses Helios and Gata3 was Helios+ Treg cells, which predominates in the small intestine and connected to IL-33-related pathways (105). In clinic, the role of microbiota in the origins of and relationships between RORγ+ and Helios+ Treg cells is still not completely understood. The strategy to manipulate the microbiota may be beneficial in inducing the particular Treg subsets to restore gut homeostasis after clarifying this mechanism. Initiating the above Tregs after microbiota colonization is the basic internal mechanism for formation and maintenance of intestinal microbe–host T cell symbiosis. After interacting with the gut microbiota, the host-naïve conventional CD4+ cells can differentiate into RORγ+ Tregs. A small proportion of conventional CD4+ cells are transformed into Helios+ Treg cells (106). PSA synthesized by *Bacteroides fragilis* is considered to be a typical commensal immunomodulatory factor. Exposure of naïve DCs to PSA induced the conversion of naïve CD4+ T cells into FoxP3+ Tregs. PSA also sends signals directly on Foxp3+ Tregs to engender mucosal tolerance through the TLR pathway (107, 108). Interestingly, PSA only originates Tregs in inflammatory states and has little effect on Tregs in a healthy environment. In contrast, *Clostridium* species elicit colonic Tregs in both healthy and inflamed states. A group of 46 species of murine *Clostridium* and 17 strains within Clostridia clusters XIVa, IV, and XVIII from a human fecal sample induced Tregs in the mouse colon LP, with consequent protection against murine colitis and allergy. However, when these strains were separately monoclonalized into GF mice, Tregs are almost unaffected, indicating that these microorganisms cooperated in Treg induction (109, 110). However, this result is challenged by many other studies. Other researchers have found that any one of the 5 Bacteroides (*B. intestinalis*, *B. caccae*, *B. thetaiotaomicron*, *B. vulgatus*, and *B. massiliensis*) led to effective colonic Treg induction (111). Geva-Zatorsky et al. also found that a variety of single strains strongly induce Tregs in the colon (48). Therefore, the difference may be caused by experimental methods, including the detailed strains and even the number of monoclonal colonizing strains.

### 2.4 Regulation of Gut Bacterial Metabolites on Adaptive Immunity

Numerous biologically active metabolites secreted by the human gut microbiome continuously interact with the host, modulate host immunity, and participate in maintaining immune homeostasis. Previous research has mainly focused mainly on short-chain fatty acids (SCFAs) (112). SCFAs are water-soluble and diffusible fermentation products of dietary fiber produced by anaerobic bacteria. They comprise one to seven carbons, with acetate, propionate, and butyrate accounting for 90%–95% of total colonic SCFAs (112). SCFAs reach a peak concentration in the cecum and decrease from the proximal to the distal colon (113). SCFAs regulate adaptive immune cell functions mainly by combining G protein-coupled receptors (GPCRs) (37, 41) and inhibiting histone deacetylase [23-26] and metabolic disturbances (32, 35, 36, 39). Among GPCRs, GPR43 recognizes a wide range of SCFAs (41), including acetate, propionate, and butyrate. Among these, acetate promoted intestinal IgA responses depending on the GPR43 pathway, which induced DCs to express Aldh1a2 and then converted vitamin A into its metabolite retinoic acid. In turn, blockade of retinoic acid signaling inhibited the acetate-induced IgA production (41). Takeda et al. showed that the bacterial metabolites, pyruvic acid and lactic acid, could induce dendrite protrusion *via* GPR31 in CX3CR1+ cells in mice, which enhanced immune response and high resistance to intestinal *Salmonella* infection (38). In addition, SCFAs are able to regulate the colonic Treg pool through Ffar2 (the gene encoding the receptor GPCR43 of SCFAs) expressed on colonic Tregs (114). Moreover, SCFAs inhibit the production of histone deacetylase, causing increased acetylation and expression of Foxp3, which is the core transcription factor that drives Treg differentiation (35, 112). Arpaia et al. found that butyrate and propionate simultaneously increase CNS1-dependent extrathymic Tregs and peripheral Tregs (32).

In addition to inducing Tregs, recent studies suggested that SCFAs also promote differentiation and functions of CD4+ T cells and CD8+ T cells, indicating that the impact of SCFAs on the adaptive immunity may be more complicated than previously thought. SCFAs, such as acetate, propionate, and butyrate, selectively regulate Th1s, Th17s, and Tregs by suppression of histone deacetylases and regulation of the mTOR-S6K pathway according to cytokine milieu and immunological context (39). It is noted that the dosage of SCFAs is vital in the specific immune effect. Kespohl et al. found that the role of butyrate on Tregs and conventional CD4+ T cells is dose-dependent. Low butyrate concentrations (0.1–0.5 mM) facilitated the amplification of Foxp3+ Tregs in the presence of TGF-β1 while at a concentration of 1 mM, the butyrate induced the colonic expression of the pro-inflammatory molecules (T-bet and IFN-γ) in Tregs and conventional T cells (112). Moreover, by promoting cellular metabolism, SCFAs effectively enhanced the memory potential of CD8+ T cells. The mechanistic experiments revealed that butyrate disassociates the tricarboxylic acid cycle from glycolytic input in CD8+ T cells, which allowed for the preferential promotion of oxidative phosphorylation through continuous glutamine utilization and fatty acid catabolism to enhance the memory potential of activated CD8+ T cells (33). Besides, systemic bacterial infections could accumulate acetate in serum within a few hours, which could be integrated by CD8+ T cell and converted into increased glycolysis and functional capacity, and further promote rapid memory CD8+ T cell response (34). It is worth noting that some effector CD4+ T cell subsets exhibited cytotoxic activity, breaking the functional dichotomy between CD4+ helper cells and CD8+ cytotoxic T lymphocytes. Histone deacetylases 1 and 2 were reported to inhibit CD4+ CTL differentiation, and SCFAs, acting as HDAC inhibitor, could upregulate CTL genes and enhance the generation of CD4+ CTLs (40).

Besides SCFAs, another bacterial metabolite, bile acids (BAs), also directly affects the adaptive immunity to maintain immune homeostasis. About 5% of BAs escape into the colon, and colonic commensal bacteria transform them into a large number of biologically active molecules. Some intestinal primary BAs (such as cholic acid, chenodeoxycholic acid, ursodeoxycholic acid) and some effective secondary BAs (such as lithocholic acid, 3-oxolithocholic acid) affect adaptive immunity through several nuclear receptors and/or GPCRs (44). As reported, 3-oxolithocholic acid can directly bind to the key transcription factor RORγt to inhibit Th17s, while lithocholic acid generates mitochondrial reactive oxygen species to increase expression of Foxp3 and the accumulation of Tregs (42, 43). Isoallolithocholic acid induces an open chromatin structure at the Foxp3 gene promoter and thus exposes the NR4A1-binding sites in this region (115), thereby leading to the activation of Foxp3 transcription to enhance Treg differentiation. 3β-Hydroxydeoxycholic acid, a secondary bile acid, regulated colonic Treg differentiation, in a conserved non-coding sequence 1 (the Foxp3 enhancer)-dependent manner. In this process, 3β-hydroxydeoxycholic acid limits the farnesoid X receptor (FXR) activity in DCs and endows DCs an anti-inflammatory phenotype, which further increases the generation of Tregs (116).

Certainly, the role of other bacterial metabolites in adaptive immunity is also gradually discovered, which included adenosine and extracellular ATP (117). The adenosine and extracellular ATP are pivotal to maintaining the function of Tregs in immune homeostasis. In the future, the coordination network of these metabolites in immune homeostasis still needs further clarification.

## 3 Gut Microbiota Regulates Adaptive Immunity Under Tumor Pathology State

**Figure 2:**
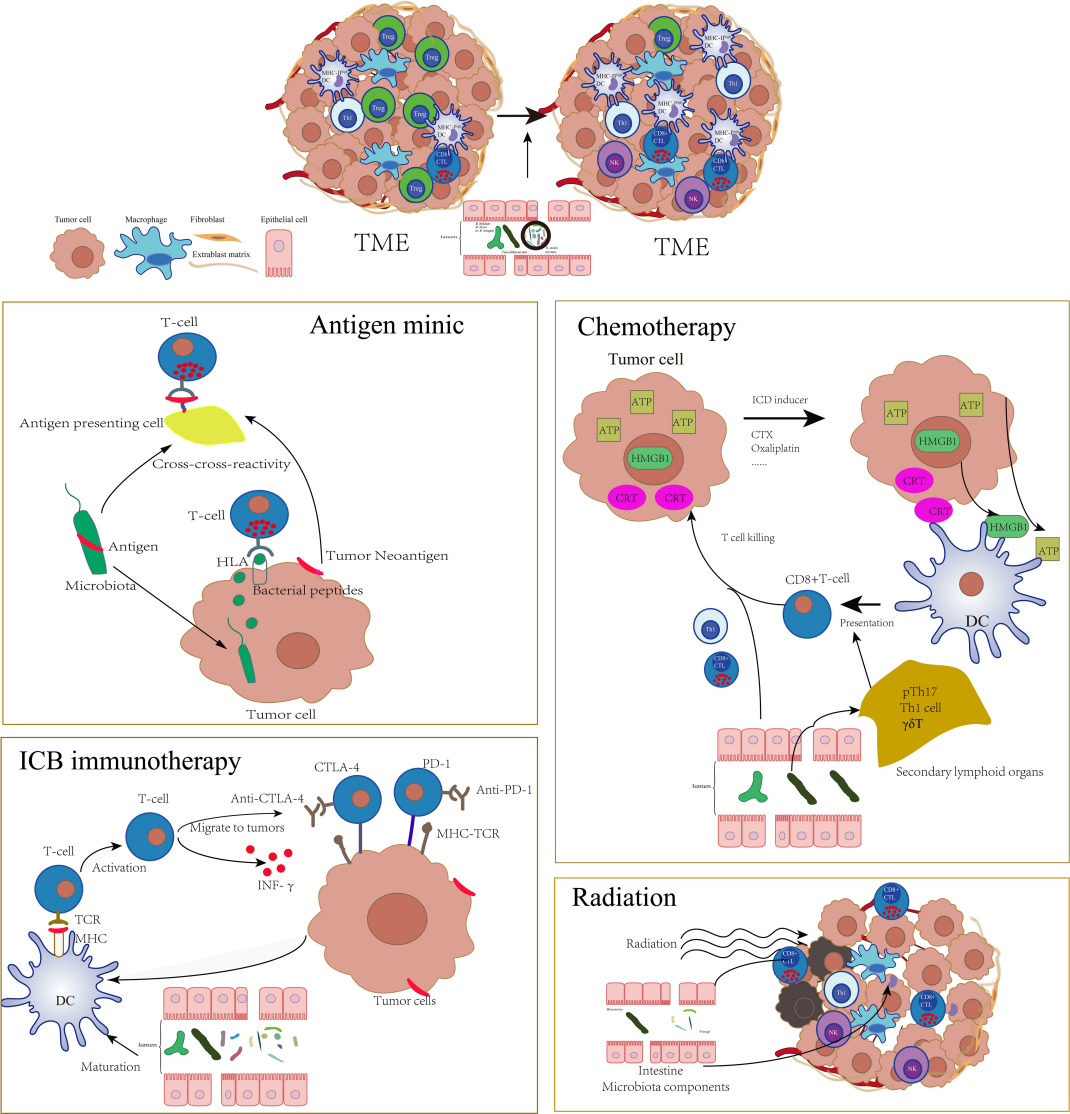
The tumor environment was profoundly altered by gut microbiota which could influence a series of antitumor therapies such as antigen mimic of microbiota, chemotherapy, ICB immunotherapy, and radiotherapy through adaptive immune approach. The specific microbiota could enhance the activation of DCs, increase the infiltration of CTLs, and decrease the Tregs in TME, which significantly sensitize these therapies.

In the tumor pathophysiological state, gut bacteria, host, and the mucosal barrier are significantly different from the immune homeostasis environment. Moreover, a series of antitumor therapies will cause the destruction of the intestinal mucosal barrier, further affect the balance of the intestinal flora (118–121), and incur the translocation of commensal bacteria to the mLN and spleen, where they stimulate the host’s adaptive immune responses and change the tone of antitumor adaptive immunity (121). As a result, the effects of a series of antitumor therapies, such as chemotherapy, radiotherapy, and immunotherapy, based on immune checkpoint blockade (ICB) targeting the PD-1/PD-L1 axis or cytotoxic T lymphocyte–associated protein 4 (CTLA-4) are profoundly altered by the gut microbiota at preclinical and clinical levels (**Figure 2**) (118, 120–129).

### 3.1 Antigen Mimic of Gut Microbiota Enhances Antitumor Immunity

High cross-reactivity is a common feature of TCR and constitutes the basic feature of T cell recognition. Mason (130) estimated that a single T cell reacts productively with approximately 10^6^ different MHC-associated minimal peptide epitopes. Bacterial-derived peptides are easier to be recognized by CD8+ T cells than peptides derived from the host itself. When a microbial epitope is similar to a tumor antigen and forms a “molecular mimic,” T cells triggered by the microbe will simultaneously recognize and attack tumor cells.

There are significant differences in spontaneous antitumor immunity in B16.SIY melanoma-bearing JAX mice and TAC mice which are in the same genetic background with different living environments (129, 131). The analysis of the significant correlation between genus-level taxa in stool and the accumulation of activated antigen-specific T cells in TME revealed that the only significant association was *Bifidobacterium(* 129 *)*. “Molecular mimic” may partially explain this association. Cross-reaction can be induced between B16.SIY tumor neoantigen and the *Bifidobacterium breve* SVY epitope*. Bifidobacterium*-rich JAX mice induce more SVY antigen-specific CD8+T cells that recognized SIY-expressing melanoma cells *in vivo* to slow down tumor growth and prolong survival time (131). Shelly Kalaora et al. also found that the bacteria that colonize melanoma tumors could enter into melanoma cells, and the bacteria-derived peptides can be presented by the HLA-I and HLA-II molecules of tumors, which ultimately modulate the functions of CD8+ and CD4+ T cell immunity (132).

The bacteriophage components carried by bacteria also provide antigen mimics. Bacteriophages are bacterial viruses that transfer immunogenic sequences to new bacterial hosts with a strict specificity. The tail length tape measure protein (TMP) of a prophage found in the genome of the bacteriophage *E. hirae* is immunogenic. *E. hirae* carrying TMP triggered TMP-specific MHC-1 restricted CD8+ T cell responses. PSMB4 is an oncogenic driver involved in proliferation and invasion in a variety of malignancies. TMP derived peptide TMP1/TSLARFANI has strong homology with the PSMB4-derived peptide. T cells recognizing the TMP1/TSLARFANI epitope cross-reacted with the PSMB4-derived peptide to enhance antitumor adaptive immunity responses. Advanced kidney cancer and lung cancer patients with detectable fecal TMP have prolonged overall survival after anti-PD-1 treatment. It is worth noting that in addition to *E. hirae*, other members of the *Enterococcus* genus can also carry bacteriophages containing TMP (133).

Although the current findings about the antigen mimic of commensal bacteria are not abundant, due to the huge number of commensal bacteria genomes and the high cross-reactivity of TCR, antigen mimic may be significant in strengthening antitumor adaptive immunity.

### 3.2 Gut Microbiota Promotes the Initiation of Antitumor Adaptive Immunity to Assist ICB Therapies

In order to trigger an effective antitumor response, tumor antigens must be taken up by DCs and presented through MHC-II and/or MHC-I to activate CD4+ T cells and CD8+ T cells, respectively. Tumor cells inhibit the recruitment, activation, and maturation of DCs by multiple pathways through the tumor microenvironment (TME) (134). Gut microbiota activates the antigen presentation and cross-presentation of DCs to sensitize the initiation of adaptive immunity (69, 120, 124, 129). Compared with patients with high abundance of *Bacteroidales* (associated with poor PD-1 immunotherapy efficacy), patients with high abundance of *Faecalibacterium* have higher density of immune cells and antigen processing and presentation markers in TME, indicating that gut microbiota are involved in the activation of tumor antigen presentation (124). In TME, infiltration of DCs with high levels of MHC-I increases after colonization of the 11-strain microbe mixture in GF mice. The accumulation of IFNγ+ CD8+ T cells is significantly impaired in MHC-Ia-deficient mice, indicating that the 11-strain mixture promoted a killing effect of tumor antigen-specific CD8+ T cells through MHC-I^+^DC-mediated cross-presentation of tumor antigens (69). In the melanoma model, the *Bacteroides fragilis*-dependent immunostimulatory effect induced by the CTLA-4 blocker depends on the mobilization of CD11b^+^ DCs in LP. These DCs stimulated by PSA of *Bacteroides fragilis* could start IL-12-dependent homologous Th1 immune responses against PSA. Adoptive transfer of *Bacteroides fragilis*-specific T cells has the ability to overcome non-response to CTLA blockers in GF mice or mice treated with antibiotics (120).

*Bifidobacterium* is widely studied as the most representative commensal bacteria. A large number of previous studies have shown that it can promote the maturation of DCs (129, 135, 136). With the popularity of immune checkpoint blockade (ICB) in recent years, *Bifidobacterium* is proved to exert antitumor adaptive immunity, spontaneously or synergistically with ICB (91, 126, 129). Stimulating the maturation of DCs is an important mechanism for *Bifidobacterium* to assist antitumor adaptive immunity. Combination of *Bifidobacterium* and the anti-CD47 monoclonal antibody facilitates DC cross-presentation through the STING pathway to upregulate CD8+T cells (137). Sivan et al. also find that *Bifidobacterium breve* and *Bifidobacterium longum* induce tumor-specific CD8+T cells in circulation and TME by promoting DC maturation. After administration of *Bifidobacterium*, the proportion of MHC-II^hi^ DCs in tumor tissues is significantly increased (129). Activation of IFN-γ signal transduction is another important way for *Bifidobacterium* to promote cellular immunity (126). The ratio of effector CD8+ T/Tregs cells and cytokine-producing IL-2+CD4+ and IFN-γ+CD8+ tumor-infiltrating T cells increased in the spleen and TME after treatment of specific *B. bifidum* strains with PD-1 treatment. Intestinal transcriptomics, serum metabolomics, and lipidomics data indicated that the synergy of specific *B. bifidum* strains to anti-PD-1 is closely related to IFN-γ, because IFNγR blockade eliminates the tumor reduction effect of anti-PD-1 plus *B. bifidum*. Furthermore, gene ontology enrichment analysis revealed that the peptidoglycan biosynthesis process is enriched in synergistic strains. IFN-γ secretion is inhibited after the peptidoglycan receptor Tlr2 is blocked, and the synergistic effect of anti-PD-1 plus *B. bifidum* is abrogated. Therefore, peptidoglycan-mediated IFN-γ signal transduction is the determinant of the strain-specific synergistic effect of specific *B. bifidum* strains on cancer treatment (126).

The diversity of gut microbiota, the types of different microbes, whether antibiotics are used, etc., profoundly affect the antitumor response and survival of patients with ICB immunotherapy (124, 126–128). Some beneficial microbiota are associated with better clinical efficacy of antitumor therapy (128). *Bifidobacterium* and *Ruminococcaceae/Faecalibacterium* have significantly higher levels in metastatic melanoma patients who respond to PD-1 blockade, by triggering antitumor immunity, such as increasing CD4+ T cells, CD8+ T cells, and memory T cells and downregulating immunosuppressive Tregs cells (124, 126, 127, 129). *A. muciniphila* is associated with the favorable outcome of PD-1 blockade in both non–small cell lung cancer (NSCLC) and renal cell carcinoma (RCC) (128). However, in another research team, the results are different. In NSCLC patients, the researchers find that *B. bifidum* is significantly enriched in responders, whereas *A. muciniphila* and *Blautia obeum* upgrade in non-responders (126). Compared with mice treated with unfavorable microbiota, mice with favorable microbiota show greater anti-PD-L1 therapeutic activity, and this benefit can be transferred through cohabitation or fecal transplantation. When transplanting fecal microbiota into GF mice, microbiota derived from patients responding to ICB treatment confers treatment response (127, 129). These indicate that good gut microbiota significantly promotes the antitumor adaptive immune response.

It is worth noting that the antitumor and antibacterial adaptive immunity stimulated by gut microbiota are independently induced in different regions. In MC38 adenocarcinoma-bearing mice, the 11-strain mixture effectively formed spontaneous or ICB-coordinated antitumor immune responses in a CD8 T cell-dependent manner, accompanied by an increase in the frequency of tumor-infiltrating lymphocytes expressing TCRs specific for MC38 tumor-associated antigen p15E. However, there is no overlap in Vβ usage between IFNγ+CD8+ T cells in colon, systemic circulation, and tumor, indicating that these IFNγ+CD8+ T cells are induced locally and independently (99). Besides, *B. breve* can mitigate colitis caused by anti-CTLA-4 without an apparent effect on antitumor immunity, through modulating the functional metabolism of Tregs (138, 139). However, there are also some bottlenecks in the future development and application in clinic, because the results of different studies are not consistent with the corresponding beneficial bacteria, indicating that specific favorable microbiota may also be related to the region, race, and other environmental factors. For instance, Routy et al. found that *A. muciniphila* was associated with a favorable clinical outcome in both NSCLC and RCC (128), while Lee showed that *A. muciniphila* was enriched in non-responsive patients with NSCLC (126). In the future, *in vitro* culture, functional analysis, and interaction mechanism of specific bacteria need to be further enriched and verified to correct the deviation of individual differences.

### 3.3 Gut Microbiota Cooperates With Chemotherapy to Exert Antitumor Adaptive Immunity

Gut microbiota also influence antitumor effects of chemotherapy through an adaptive immune approach. As we know, humoral immunity and T-cell immunity are incompletely reconstituted in years following mostly intensive cytotoxic chemotherapy in cancer patients (140). A large number of studies focused on the dosage and regimens of chemotherapy, hoping to reduce the impact of chemotherapy on the adaptive immune system as much as possible, such as metronomic regimens without interrupting key immune players in the antitumor responses (141). During the interaction between chemotherapeutic drugs and host, microorganisms also play a role in regulating the impaired adaptive immunity and enhanced antitumor responses. Besides, a portion of chemotherapeutics as a producer of immunogenic cell death (ICD) could elicit adaptive immune responses and trigger T cell-dependent immunity themselves, such as cyclophosphamide, thiostrepton, oxaliplatin, and anthracyclines (142). Hence, the gut microbiota may also act as sensitization agents for better chemo-immunotherapy effect. For instance, Daille’re et al. found that *Enterococcus hirae* and *Barnesiella intestinihominis* are necessary for the antitumor effect of cyclophosphamide (CTX) (118). *E. hirae* can partially restore the anticancer Th1 and CTL responses induced by CTX in mice after the treatment with broad-spectrum antibiotics. In secondary lymphoid organs, *E. hirae* induces CD8+ T cells and pTh17s, while *B. intestinihominis* motivates CD8+ T cells and Th1s. All these induced immune responses by *E. hirae* are associated with longer progression-free survival in advanced lung and ovarian cancer patients treated with chemo-immunotherapy (118). Besides, oxaliplatin-induced cell death of ileal EC inversely governs the immunogenic *Erysipelotrichaceae* and tolerogenic *sobacteriaceae* proportions in the ileum, dictating the balance between antitumor Tfhs and deleterious Th17 responses in colon cancer (118, 143). Moreover, compared with oxaliplatin alone, oxaliplatin plus specific *Bifidobacterium* strains (*B. bifidum* KCTC3357 and *B. bifidum* KCTC3418) significantly increase the number of antitumor lymphocytes, including effector CD8+ T cells and the ratio of CD8+ T/Tregs cells in spleen and tumors (126). These results all revealed the contribution of intestinal bacteria in restoring the adaptive immunity of chemotherapy.

In addition to the beneficial antitumor immune responses, the gut microbiota also contributed to the side effect caused by chemotherapy *via* influencing immunity. Pyter et al. found that paclitaxel induced the increase of intestinal permeability and triggered the immune system to aggravate neuroinflammation (144). They preliminarily found that the decreased cognitive performance occurred in parallel with reduced microglial immunoreactivity and transient increases in pro-inflammatory cytokine/chemokine (Il-1β, Tnfα, Il-6, and Cxcl1) gene expression in the brain, which may be mediated by the brain–gut–microbiota axis (145, 146). More studies like this may be helpful for decreasing the side effects of chemotherapy.

### 3.4 Gut Microbiota Cooperates With Radiotherapy to Exert Antitumor Adaptive Immunity

Compared with immunotherapy and chemotherapy, the immune relationship of gut microbes and radiation therapy (RT) still remains mostly unknown. As reported, a high density of tumor-infiltrating CTLs, decreased myeloid-derived suppressor cells, and decreased tumor-associated macrophages correlates with a favorable clinical outcome for RT (147). Shiao et al. indicated that bacterial depletion with antibiotics reduces the efficacy of RT in murine models of breast cancer and melanoma (4), while fungal depletion caused an increase in RT efficacy, which revealed the presence of fungi which seems to contribute to the generation of a suppressive tumor microenvironment to disturb the effect of RT. Interestingly, Riquelme et al. revealed that fungi *C. albicans* overgrowth increases exhausted CD8+ T cells and ultimately impairs the antitumor response to RT and decreases survival (148). An interplay between innate and adaptive immunity is implicated and orchestrated by Dectin-1, a member of the C-type lectin family of pattern-recognition receptors, which is expressed in breast cancer tissue to sense pathogens present in the fungal cell wall to activate the antifungal protective immune pathway (149). Hence, strict control of microbiota components may be pivotal to guaranteeing the effective response of RT or overcoming the resistance of RT.

### 3.5 Active Metabolites of Gut Microbiota Participate in Mediating Antitumor Adaptive Immunity

Under the pathophysiology of tumors, with changes of gut microbiota and damages of the mucosal barrier, the type, ratio, and concentration of microbiota-derived metabolites change accordingly in intestinal tissues and systems. These altered metabolites remold antitumor adaptive immunity and even influence antitumor efficiency.

The types of SCFAs in the metabolites of different commensal bacteria are different. Most commensal bacteria produce large amounts of acetate and formate, while some produce propionate. *Faecalibacterium prausnitzii* and *Anaerostipes hadrus* generate large amounts of butyrate. It is noted the dominant commensal bacteria in gut cannot produce pentanoate. As pentanoate is a rare bacterial metabolite, the low-abundance commensal bacteria *Megasphaera massiliensis* is the only bacterium known to synthesize high amounts of pentanoate (113). *In vitro* treatment of CTL and CAR-T cells with valerate and butyric acid can increase the function of the mTOR pathway, induce metabolic changes, and inhibit HDAC-I. The reprogramming increases the production of effector molecules such as CD25, IFN-γ, and TNF-α that significantly enhanced the antitumor activity of antigen-specific CTL and CAR-T in cancer models (113). In addition, after oral administration of inulin, which eventually increases the concentration of SCFAs, the antitumor adaptive immune phenotypes including circulating antigen-specific CD8+ T cells and CD44+CD62L+CD8+ memory T cells were significantly increased, further confirming the existence of a long-term memory response (146).

Consistent with the above studies, data of clinical observations also indicate that fecal SCFA concentrations may be associated with PD-1 efficacy. In a cohort study of 52 patients with solid tumors and another clinical study from 11 cases of NSCLC, high concentrations of fecal and serum SCFAs are associated with positive responses to anti-PD-1 treatment and longer progression-free survival (150, 151). However, the role of SCFAs in tumor immunity is multifaceted and even ambiguous. In metastatic melanoma patients and mouse models, SCFAs (butyrate and propionate) limit the antitumor effects of CTLA-4 inhibitors *via* inducing CTLA-4 blocking resistance and a high proportion of Tregs. In addition, high blood butyrate levels attenuate the accumulation of memory CD4 + T cells and IL-2 impregnation induced by CTLA-4 blockade (152). Yang et al. also found that gut microbiota-derived butyrate inhibited STING-activated type I IFN expression in dendritic cells (DCs) through blockade of TBK1 and IRF3 phosphorylation, which suppressed ionizing radiation-induced tumor-specific cytotoxic T cell immune responses (153). All these findings indicated that selective targeting of SCFA-producing microbiota may provide a novel option to enhance tumor therapies sensitivity.

Inosine is also an active metabolite of bacteria, which mediates the antitumor effect of *Bifidobacterium pseudolongum* combined with ICB in the treatment of colorectal cancer (CRC) models. The role of inosine in inducing or inhibiting the differentiation of Th1s proved to be dependent on the specific microenvironment. In the presence of IFN-γ, inosine-A2AR-cAMP-PKA signaling cascade leads to phosphorylation of the transcription factor cAMP response element-binding protein (CREB), a known transcriptional enhancer of Th1s differentiation factors. While without IFN-γ, inosine inhibits Th1 differentiation [80].

## 4 Conclusions

Whether in the context of immune homeostasis or tumor pathophysiology, intestinal commensal microorganisms regulate host adaptive immune responses in multiple dimensions through multiple pathways (12, 21, 154, 155). Commensal bacteria are the most important founder of immune homeostasis and an important factor affecting antitumor immunity, especially immunity-related treatment (21). In recent years, important progress has been made in exploring the mechanism of the gut microbiome-antitumor adaptive immune system axis and characterizing the impact of the microbiome on tumor immunobiology (12, 21, 154, 155), but the specific details are still elusive to a large extent. How does the flora-specific adaptive immunity caused by microbiota disturbance promote tumor-specific immunity? How can specific flora regulate or even reverse the tumor immunosuppressive microenvironment? The role of the active metabolites of the flora is diverse and may play diametrically opposite effects under different backgrounds and conditions. It is necessary to further clarify the specific background and conditions under which active metabolites promote antitumor immunity, as well as the decisive factors affecting these backgrounds and conditions. In addition, stratification at the species or strain level is important because microorganisms within the same genus may have different effects on the same disease process, which makes it difficult to define a universal “good” microbial group based on composition.

Because gut microbiota has greater genetic diversity than the host and can be manipulated, it is an urgent need for antitumor therapy to achieve precise stratification and precise regulation of tumor immunotherapy through precise cognition of gut microbiota. Therefore, it is very valuable and promising to further explore the regulation mechanism of gut microbiota on adaptive immunity in a tumor pathological environment and specific antitumor therapies. In this field, as adaptive immunity is a complex network, a major challenge will be to understand the coordination degree between the microbiota and many types of immune cells on immunity and even immune tolerance, which include the cells in lymph, stroma, endothelium, and epithelium.

## Author Contributions

YL, LM, and ZY initiated and conceived this review. ZY, YL, JZ, and SF participated in selecting and reviewing literature data. YL and LM performed analyses based on the literature and wrote the literature. LM and CZ supervised and revised the manuscript. All authors contributed to the article and approved the submitted version.

## Funding

The work was supported by grants from the National Key Research and Development Project of China (No. 2019YFC1605800).

## Conflict of Interest

JZ and SF were employed by Wecare Probiotics Co., Ltd.

The remaining authors declare that the research was conducted in the absence of any commercial or financial relationships that could be construed as a potential conflict of interest.

## Publisher’s Note

All claims expressed in this article are solely those of the authors and do not necessarily represent those of their affiliated organizations, or those of the publisher, the editors and the reviewers. Any product that may be evaluated in this article, or claim that may be made by its manufacturer, is not guaranteed or endorsed by the publisher.
